# Diet-derived microRNAs: unicorn or silver bullet?

**DOI:** 10.1186/s12263-017-0564-4

**Published:** 2017-06-22

**Authors:** Kenneth W. Witwer, Chen-Yu Zhang

**Affiliations:** 1 Departments of Molecular and Comparative Pathobiology and Neurology, Johns Hopkins University, Baltimore, USA; 2 School of Life Sciences, Nanjing University, Nanjing, People’s Republic of China; 3 Jiangsu Engineering Research Center for MicroRNA Biology and Biotechnology, State Key Laboratory of Pharmaceutical Biotechnology, School of Life Sciences, Nanjing University, Nanjing, China

**Keywords:** sRNA, Cross-kingdom, Genetically engineered, Biotechnology, miRNA, Diet, Microbiome, miRNA, Plan

## Abstract

In ancient lore, a bullet cast from silver is the only effective weapon against monsters. The uptake of active diet-derived microRNAs (miRNAs) in consumers may be the silver bullet long sought after in nutrition and oral therapeutics. However, the majority of scientists consider the transfer and regulation of consumer’s gene activity by these diet-derived miRNAs to be a fantasy akin to spotting a unicorn. Nevertheless, groups like Dr. Chen-Yu Zhang’s lab in Nanjing University have stockpiled breathtaking amounts of data to shoot down these naysayers. Meanwhile, Dr. Ken Witwer at John Hopkins has steadfastly cautioned the field to beware of fallacies caused by contamination, technical artifacts, and confirmation bias. Here, Dr. Witwer and Dr. Zhang share their realities of dietary miRNAs by answering five questions related to this controversial field.

## What is the best evidence available, pro and con, for the significant uptake, distribution, and clearance of exogenous miRNAs from animal or plant sources that is of potential functional relevance?

### Ken Witwer answers

The best evidence to date pertaining to questions of xenomiR uptake derives from transgenic animal studies. In animal models, endogenous RNAs can be manipulated, and exposures can be completely controlled and monitored. In the first such study [1], miR-21 knockout mice received a diet replete with miR-21 but showed no evidence of substantial uptake. In another study, genetically modified mice were used to alter the exposure of pups to miR-30b in milk [2]. No difference in miRNA levels was observed in pups that received milk from nursing animals with elevated versus normal levels of miR-30b. As a third example, pups from two miRNA knockout models (miR-375 and miR-200c/141) showed no evidence of miRNA uptake from milk of wild-type animals despite high levels of miRNAs in milk [3]. Together, these studies provide strong evidence against biologically meaningful uptake of dietary xenomiRs by adults or pups, even when conspecific miRNAs, miRNA-binding proteins, and miRNA-protective vehicles such as lipid vesicles or other particles are involved. It should be noted that although the authors of these studies used sensitive and ligation-independent quantitative polymerase chain reaction (qPCR) assays, it remains possible that low levels of uptake occurred at or below the limits of detection. However, any such uptake remained orders of magnitude below commonly accepted copy number thresholds in the cell [3–5].

In contrast, positive reports of uptake and function have been marked by apparent artifact. An initially exciting and influential report of plant MIR168a uptake and function [6, 7] now appears to rest on data consistent with contamination, not uptake [8]. Similarly, the reported function in the initial study [6, 7]—regulation of a single gene involved in cholesterol homeostasis—has been revealed as artifactual, a misinterpretation owing to lack of a crucial dietary control [9]. Elsewhere, highly efficient uptake of MIR528 was reported in humans after ingestion of 3 l of watermelon juice [10, 11], yet watermelon, a dicot, does not encode the monocot-specific MIR528 [12]. The challenges of spurious detection and contamination in foreign nucleic acid studies cannot be overstated [6, 7, 13–16] and are not limited to cross-kingdom communication. For example, purported uptake of milk miRNAs by mammals [17] could not be reproduced by a different laboratory using the same samples [18]. To the extent that bona fide detection of transferred dietary miRNAs occurs, there is often a startling disconnection between the concentrations observed in vivo (if they can be credited) and those used in functional experiments. In a typical experimental workflow, seeming detection of vastly subhormonal (e.g., attomolar) levels of xenomiRs in vivo are followed by nonphysiologic (e.g., nanomolar) transfection experiments [19]. These levels are many orders of magnitude above what could be reached in vivo [20]. Importantly, xenomiRs do not appear to associate with the host regulatory machinery [21–23]; thus, canonical function would not occur even in the improbably event that otherwise regulation-relevant levels could be reached.

### Chen-Yu Zhang responds

The mobility of small RNA molecules (siRNAs and miRNAs) from one species to another is a newly discovered mechanism for cross-talk between different organisms, even between species of different kingdoms. Transfer of double-stranded siRNA has been frequently reported to occur between closely interacting pathogenic, parasitic, or symbiotic organisms [24–28]. Single-stranded miRNA was also found to be transferred between host and invader [29–31]. A highly debated issue that has not yet been resolved convincingly is whether there is the transfer of small RNAs between complex organisms. To date, the best evidence for the significant uptake and distribution of functional exogenous miRNAs comes from the observation of plant miRNA-mediated cross-kingdom regulation. In 2012, we reported a previously uncharacterized phenomenon: ingested plant miRNAs can pass through gastrointestinal tract, enter the peripheral blood stream, accumulate in tissues, and exert gene regulation in mammals [6, 7]. Our follow-up study further showed a kinetic absorption curve of dietary plant miRNAs: when volunteers are fed watermelon juice and mixed fruits, six out of 16 selected miRNAs showed a dynamic physiological pattern in their plasma with an absorption rate of 0.04 to 1.31%; the levels of dietary plant miRNAs peaked within 3–6 h after intake in serum and tissues [10, 11].

Independent studies have provided the evidence both for and against the dietary miRNA uptake by mammals. Two studies reported little or low measurable uptake of plant miRNAs by PCR in human and primates after a plant food-feeding study [1, 16]. Dickenson et al. attempted to validate our original research but found little dietary uptake of miR168a or downregulation of LDLRAP1 by miR168a after rice feeding [9]. For the contradictory detection of plant miRNA uptake from the diet, we have emphasized several critical issues to be carefully considered, such as the selection of proper miRNAs, accurate normalization, suitable RNA isolation method, and minimized sequencing bias (for more details, see our replies) [10, 11, 32]. On the other hand, some evidence suggest dietary miRNA is a real physiological phenomenon. A group showed that dietary miRNA can survive for 36 h or longer in tissues; specifically, the level of MIR172 was approximately 4.5–0.4% (2–24 h after feeding) in the stomach, 2.4–0.2% (2–36 h) in the intestines, 1.3–0.2% (2–72 h) in the blood, and 0.38–0.04% (2–72 h) in the spleen [33]. Beatty et al. [34] identified abundant nonhuman small RNA sequences derived from dietary plant material in plasma and exosomal fraction. Yang et al. [21–23] were able to detect MIR2911 and MIR168a from the sera of mice fed a chow diet containing honeysuckle and synthetic MIR168a. The plant miRNA levels decreased to background levels after the honeysuckle diet was replaced with a normal chow diet, proving that the detected miRNAs are absorbed from food. Further works by Yang et al. [21–23, 35, 36] suggested that MIR2911 exhibited unusual stability, was not associated with exosomes or the Argonaute complex during circulation, and had the stability which may be conferred by modifications from the host.

Meanwhile, some experimental results have provided direct evidence for the active function of dietary plant miRNA in animal consumers in multiple areas, including metabolism, viral infection, immune responses and cancer. Our follow-up study showed that MIR2911 from a honeysuckle decoction has an anti-viral effect against influenza A viruses including H1N1, H5N1, and H7N9 (Zhou [37]). Chin et al. reported that plant MIR159 was predominantly detected in Western human sera, with the abundance of this miRNA in the serum being inversely correlated with breast cancer incidence and progression in patients; they further showed that oral administration of a MIR159 mimicked significantly suppressed the growth of xenograft breast tumors in mice by targeting TCF7 [19]. Mlotshwa et al. [38] showed that oral administration of tumor suppressor miRNAs reduced tumor burden in a mouse colon cancer model, suggesting that engineered plant-expressing artificial miRNAs can be used as dietary miRNA drugs to treat human cancers. Interestingly, food-derived plant miRNA could also function in recipient cells in a sequence-independent manner: evidence showed plant miRNA could dampen inflammation by binding to toll-like receptor 3 (TLR3) of dendritic cells [39].

The studies of animal miRNA uptake also meet various challenges. While plant miRNAs in animals can be accurately measured because of the sequence difference between plant and animal miRNAs and the specific 2′-*O*-methylation in the 3′ ends of plant miRNAs, animal miRNAs derived from food are more difficult to measure because of the high sequence conservation that obscures differences between dietary and endogenous miRNAs. Baier et al. [17] first showed that humans absorb biologically meaningful amounts of miRNAs from nutritionally relevant doses of cow’s milk. However, some follow-up studies obtained contradictory results. For example, Snow et al. [1] conducted several experiments on miR-21 null mice but were unable to detect robust level of dietary miR-21 in mice consuming miR-21. One explanation here is the possible selective absorption of dietary miRNAs by mice. The sequence, nucleotide composition, modification, packaging, and protein association of dietary miRNAs all contribute to the efficacy of uptake, yet the exact mechanisms are still unclear. For example, intrinsic stability conferred by the nucleotide sequence and composition can determine dietary miRNA absorption. To our knowledge, MIR2911 shows significant uptake because of its unique sequence and high GC content, leading to high stability. Disruption of the MIR2911 sequence by just two GC nucleotides abolishes its stability and absorption (Zhou [37]). The structures that miRNAs packed in may be also responsible for selective dietary miRNA absorption. Thus, miRNA abundance is not the sole determinant of dietary miRNA uptake, and certain miRNAs enriched in food may remain undetectable. Because the possible selective absorption of dietary miRNA, randomly picking one or two plant miRNA to measure the dietary miRNA uptake in animal is highly risky. Exactly what sequence arrangement or nucleotide composition may be accessible? What type of miRNA modifications could produce high efficacy of uptake and functionality of dietary miRNAs? These issues still remain to be addressed in the future.

### Ken Witwer concludes

While the Zhang group’s astute summaries, above, of their previous claims and those of several others are helpful and appreciated, they do not address my points or other critiques in the peer-reviewed literature questioning the methodology and conclusions of these studies. The link of putative MIR168a uptake to LDL levels [6, 7] has been refuted at the level of RNA uptake [1, 9, 16] and function [9], with the initial results attributed to sequencing artifact [8]. The reportedly efficient uptake of a specific miRNA from watermelon [10, 11]—a miRNA that apparently does not exist in watermelon [12]—raises questions about the interpretation and reliability of that feeding study. The finding that MIR2911 is not a microRNA and does not associate with miRNA-related regulatory machinery [21–23] suggests that effects attributed to this sequence [40] may be anomalous phenomena that on the one hand may be worthy of follow-up but on the other have no obvious relationship to canonical miRNA mechanisms. The quadrillion-fold difference between observed circulating levels of the xenomiR MIR159a and experimental dietary exposure [19] highlights the experimental gap between the “dream” and “reality” (to borrow language from a recent review [14]) of xenomiR-mediated regulation, which I cover in Question 2, below. Finally, while one study [38] claimed validation of the Zhang results, on closer examination, it did not. In this study, xenomiR levels in circulation were not monitored; uptake into tissue was not demonstrated directly; the relative levels of foreign and endogenous miRNAs were not compared; only one in three gavaged miRNAs could be detected in gut contents after a single wash; well-known targets of the suppressor miRNAs were not measured; there were no controls for individual miRNA exposure; and effects on innate immune mechanisms (including Toll-like receptor activation [41]) were not monitored. The milk feeding results of Baier et al. [17] could not be confirmed in a replication study [18] using the same samples or even—importantly—in analysis of sequencing data from the same group [18]. In none of the negative studies that now challenge the xenomiR function hypothesis were one or two miRNAs picked at random, as the Zhang group suggests. Instead, study design focused on the same miRNAs that had previously been reported as absorbed and/or functional. Rather than shifting the goalposts—whether from miRNAs to ribosomal fragments or other non-coding RNAs or from general uptake to hypothetical sequence-specific mechanisms—we should first concentrate on independent replication of the basic observations. Of course, this has been done, with negative results. Even as I finalized this conclusion, yet another investigation of multiple datasets from multiple organisms emerged [42], in which the two most widely mapped plant xenomiR sequences were members of the MIR168 and MIR156 families, yet, curiously, MIR168a was found even in single-celled organisms from lab cultures that were not exposed to plants [42], confirming the repeated observations [6–8] that detection of this sequence in foreign organisms is artifactual. Evidence was also presented for the artifactual nature of MIR156 detection [42]. These outcomes are disappointing to all of us who were or are enthused by the xenomiR hypothesis, but we ultimately must follow the data.

## What are the gaps in experimental functional studies of exogenous miRNA and the most significant challenges to addressing them successfully?

### Chen-Yu Zhang answers

There are two types of “exogenous miRNAs,” including absorbed dietary miRNAs and endogenous miRNAs secreted by animal tissues (to the recipient cell/tissue, those secreted miRNAs are “exogenous”). It is important to emphasize our working model of exogenous dietary miRNA in adult animal that free dietary miRNAs are absorbed by epithelial cells in the GI tract where those dietary miRNAs are then packaged into exosomes and released via exocytosis. Consequently, exosome-encapsulated dietary miRNAs are delivered into recipient cell/tissue where they block the expression of target genes in a working manner of endogenous secreted miRNA. Actually, the functional studies of cross-kingdom regulation of dietary miRNA are technically quite simple to performance both in vitro and in vivo, and many groups have already confirmed it independently. Moreover, the detection of absorbed dietary miRNA is also preformed and successfully detected by many groups independently.

Therefore, the gaps in experimental functional studies of exogenous dietary miRNA are to understand the mechanism of absorption and to appreciate the robust biological activity caused by such low level of secreted miRNA (including endogenous secreted miRNA and absorbed dietary miRNA). The most significant challenge to address them successfully is to figure out the mechanism of absorption of dietary miRNA (indeed, we have already identified a membrane transporter to uptake mature miRNA). It is also helpful to understand the whole picture of exogenous miRNA by further studying the mechanisms of exosome packaging, secretion, and function in recipient cell.

### Ken Witwer responds

One important gap in our understanding, as Dr. Zhang astutely intimates, is the gap between observation and experimentation, which I also mentioned above. For example, in one rodent feeding experiment, animals were fed with small RNAs at a level approximately 16 orders of magnitude above what was initially detected in vivo [19] (for more discussion, see [20]). That such massively disproportionate amounts of xenomiRs are needed to observe effects on biology effectively refutes xenomiR functionality. It should be incumbent on those who impute function to miRNAs at vastly subhormonal concentrations to demonstrate how such action could occur in physiological systems.

Another gap is what I would call the packaging problem: There seems to be an assumption in much of the xenomiR literature that mature xenomiRs float about freely (whether in or outside extracellular vesicles (EVs)), enter cells (via transporters or promiscuous EV cell fusion), and readily integrate into host Argonautes (AGOs)/RNA-induced silencing complexes (RISCs) to regulate endogenous transcripts. But do free small RNAs exist in biological systems? The mature, single-stranded siRNA is made after being loaded in double-stranded precursor form into AGO and does not appear to transfer between AGOs [43]. A free, single-stranded small RNA is “dead”: subject to immediate degradation in vivo [44] and divorced from effector proteins including AGO. How would a plant xenomiR survive the mammalian system if parted from its AGO? Or, if still protected by plant AGO, how would it be imported into the cell as a complex and plugged into a foreign RISC? To be sure, massive molar excess might yield results. At micromolar concentration, synthetic RNA can be taken up into some cells through a process known as gymnosis [45]. Excess exogenous single-stranded small RNAs might even bind to non-AGO proteins or be incorporated into AGO. But now, we are back to the concentration gap between controlled experimental possibilities on the one hand and biological reality on the other. The packaging issue is addressed in greater depth elsewhere [5].

### Chen-Yu Zhang concludes

As I have mentioned above that the mechanism of dietary small RNA absorption is a critical issue to understand our observation. Our recent transporter study (mentioned above, paper under review) supports the working model that dietary single-strand microRNA is absorbed by epithelial cells in the mammalian GI tract and then is packaged into exosomes, importantly, with host cell RISC complex. At this stage, the absorbed dietary microRNA is already became “endogenous secreted microRNA” [6, 7, 10, 11, 40]! Our study has demonstrated that exogenous circulating plant microRNAs are generally enriched in mammalian cell exosome [6, 7, 10, 11, 37, 40]. On the other hand, it seems to me that Dr. Witwer confused the absorption rate of synthetic small RNA to that of natural plant microRNA in food. In fact, we have clearly pointed that the absorption rate of synthetic microRNAs through mouse GI tract is much less than that of natural food microRNAs (MIR-168, 4000 times; MIR-2911, 1800 times, respectively ([6, 7, 10, 11], Zhou [37]). Moreover, we and other groups have reported that recovery rates of certain dietary microRNAs in the blood are from 0.04 to 1.31% ([6, 7, 10, 11], Zhou [37]) and 1.3% [34]. It is for sure that mechanism underneath different absorption of synthetic microRNA and natural dietary small RNA requires further study.

## What evidence, pro and con, is available for indirect health effects in humans of exogenous miRNA, e.g., on the human gastrointestinal microbiome?

### Ken Witwer answers

To my knowledge, no published studies to date have demonstrated an effect of dietary xenomiRs on human health, whether directly or indirectly through modulation of gastrointestinal microbes. However, there is evidence that dietary nucleic acids in general affect the gastrointestinal microbiome as a source of nutrition that may be exploited to varying extents by different microbial communities [46]. In the mammalian gut, nucleases, proteases, and lipases act to break down nucleic acids as well as proteins and lipids that might otherwise protect DNA or RNA. Phosphatases and nucleosidases convert nucleotides into nucleosides and bases (purines and pyrimidines). These breakdown products are then imported by enterocytes: nucleotides can be recycled directly, while other products enter salvage pathways. The same products are also used by bacteria. Nucleotides in food sources have been found to promote growth of “beneficial” bacteria such as *Bifidobacterium* both in vivo [47] and in vitro [48]. It is thus not surprising that a large influx of foreign nucleic acids would stimulate growth of microbial communities, whether in the gut [47] or in soil [48]. It remains incompletely understood why different bacteria, e.g., *Bifidobacteria*, appear to respond differently to environmental nucleic acid breakdown products.

The effects of exogenous nucleic acids on microbes appear to be chiefly nutritional, not informational. There is no evidence that defined foreign nucleic acid sequences, such as those of individual xenomiRs, have specific, direct effects on the microbiome. To be sure, the authors of one recent publication [47] have advanced the hypothesis that host miRNAs (not xenomiRs) affect microbes directly. This is an intriguing concept, since the host-microbe interaction would be subject to relevant co-evolutionary pressures. Several lines of evidence were presented for miRNAs affecting microbes [47]. Alternative interpretations are possible, though. Knocking out all miRNA production in the gut, as was done [47], may have effects on gut physiology that might explain the reported findings. As we have seen above, adding large quantities of RNA to bacteria, as was also done, has non-specific nutritional effects. Finally, it is unclear that the reported effects would be achieved at physiologic, dietary concentrations of nucleic acids. Even gavaged, chemically modified miRNAs are wasted on animal model studies, as they are either undetectable or variably detected just above background even after 30 days of gavage at quadrillions of copies per day [38]. Much more work would be needed to interrogate possible functional uptake of xenomiRs by microbes. Happily, since this field is only in its infancy, its practitioners will have the opportunity to avoid pitfalls from mammalian miRNA studies, including drastically nonphysiologic and improperly controlled experiments [20].

### Chen-Yu Zhang responds

Indeed, investigation of the uptake and functions of dietary exogenous miRNAs is just at its infancy. Recent studies suggest that dietary plant miRNAs have a functional impact on consumer organisms in a cross-kingdom manner. We first showed that food-derived MIR168a can bind to target gene LDLRAP1 and reduce its expression, leading to elevation of mouse blood LDL levels. Our follow-up study identified MIR2911, a honeysuckle-encoded atypical miRNA, as the most stable miRNA in honeysuckle decoction. Since honeysuckle is a well-known Chinese herb used for the treatment of influenza A virus infection, we showed that MIR2911 in the honeysuckle decoction directly suppressed various influenza A viruses, including H1N1, H5N1, and H7N9 both in vitro and in vivo (Zhou [37]). Chin et al. [19] found that Western women sera contained plant MIR159 and that its abundance was inversely correlated with the incidence and progression of breast cancer in patients. They further showed that oral administration of a MIR159 mimicked significantly suppressed the growth of xenograft breast tumors in mice by targeting TCF7. Taking advantage of food-derived miRNA as a new therapeutic strategy, Mlotshwa et al. [38] engineered plants to express artificial tumor-suppressing miRNAs for cancer treatment in a mouse model. Pastrello et al. [49] confirmed the presence of plant miRNAs in human blood and suggested that miRNAs cooperate with other Brassica-specific compounds in a possible cancer-preventive mechanism. In addition, Cavalieri et al. [39] found that plant miRNAs can serve as a novel form of immunomodulatory agents. They showed that plant miRNAs modify the capacity of dendritic cells to respond to inflammatory agents by limiting T cell proliferation. This immunomodulatory effect was dependent on plant miRNAs binding to TLR3 and impairing TRIF signaling. This study indicates that exogenous plant miRNAs may serve as a ligand and exert biological function at a relatively low concentration. In the light of these findings, exogenous plant miRNAs may also have indirect effects on human health from many aspects.

### Ken Witwer concludes

Since the response does not address the host microbiome but instead repeats claims about other systemic functions of xenomiRs on the host, I limit my conclusion to three observations. First, as stated previously, MIR2911 is neither a miRNA nor honeysuckle-specific: it is a sequence included in part or in whole in length-polymorphic fragmentation products of 26S ribosomal RNA, which is conserved across the plant kingdom. There is no evidence that honeysuckle contains more (or more potent) 26S rRNA degradome sequences than potato, maize, nightshade, and etc. Indeed, the sequence recognized by the commercial “MIR2911” qPCR assay used by Zhou et al. [40] differs by a nucleotide from the reported honeysuckle sequence. Second, Mlotshwa et al. [38] did not feed mice with plants engineered to express anti-tumor miRNAs, as is stated; instead, they gavaged large quantities of synthetic, modified RNA in exposures that could not be achieved through plant feeding, and with unclear results, as mentioned above. Third, the qPCR detection method of Pastrello et al. [49] could not have yielded meaningful results as reported, since the specified amplification primers were designed to the same strand of the cDNA reverse transcription product. Because of this and other apparent problems that I and others have identified in public commentary (see https://www.ncbi.nlm.nih.gov/pubmed/27604570#cm27604570_30577 and https://www.ncbi.nlm.nih.gov/pubmed/27604570#cm27604570_30673), I would recommend that interpretation of this study be withheld until the authors and editors have responded to the post-publication critiques^1^.

## Why the persistent interest on putative health effects of exogenous miRNA?

### Chen-Yu Zhang answers

There are three possible explanations: (1) People normally is in fear of new thing they have been unaware or have not understood, especially if the new finding is “extraordinary” or contradictory to the conventional concept, for example, European people were afraid of tomato 300 years ago when tomato was firstly imported from America; (2) the positive effect of dietary miRNA has not been appreciated and advertised, for example, plant miR2911 in honeysuckle soup can directly shut down influenza viruses, including H1N1, N5N1, and H7N1 in vivo; (3) people will appreciate the finding of dietary miRNA absorption and cross-kingdom regulation when they realize that we can make a new type of “medical food” to treat various diseases.

### Ken Witwer responds

I agree completely with Dr. Zhang’s point number 1. Interest in the putative health effects of xenomiRs may indeed be rooted in part in fear of the unknown or new discoveries. The idea of general dietary xenomiR function, despite having now been largely refuted, has been seized upon by some to question the safety of biotechnological innovations such as targeted crop engineering. Yet humans are exposed, with no known consequence, to a great diversity of dietary plant small RNAs with homology to human transcripts [50], and even sequences engineered to target an essential mammalian gene had no apparent effect in rodent experiments [51]. These results strongly support the safety of dietary RNA (but further contest xenomiR functionality). Thus, on points 2 and 3, I must respectfully disagree. MIR2911 is not a miRNA but rather a length-polymorphic degradation fragment of a ribosomal RNA found across the plant kingdom. No longer classified as a miRNA by miRBase, MIR2911 is not specific to honeysuckle and consists almost entirely of guanines and cytosines, which may complicate accurate detection and mapping and lead to aggregates that are relatively resistant to degradation. The Hirschi lab has reported that whatever the source of “MIR2911” qPCR signal, it is not associated with AGO (20), or with EVs or proteinase-K-sensitive complexes [35, 36]. Thus, it is difficult to credit the concept that MIR2911 could silence host or viral transcripts through canonical RNA silencing, as interesting as Dr. Zhang’s influenza data surely are.

In my view, interest in xenomiR function in health continues today mostly because the concept is so compelling, fresh, and revolutionary—even if it is ultimately unfounded. It is a concept we naturally want to prove. That one of the most labile components in food (and who does not love food?) could have drastic effects by communicating with the body, at practically homeopathic levels, explaining why some foods are better than others, is truly a fascinating idea. Interest has been further bolstered by real and perceived funding and entrepreneurial opportunities: grant programs and opportunities from public sources and also various industry funding groups that sponsor research into the intriguing idea of “functional food.” Finally, around the world, there is a financial interest in scientifically underpinning so-called traditional or complementary medicine remedies, which are typically not regulated in the same way as pharmaceuticals and thus may offer profit opportunities.

### Chen-Yu Zhang concludes

Although Dr. Witwer and I both agree that putative health effects of exogenous miRNA is not as serious as some people suggested, we are considering from different aspects. Dr. Witwer believes that absorption of dietary microRNA is not real, and thus, it should not effect on consumer, neither good nor bad. While I tend to reap the benefit of this discovery, for example, we have generated RNAi transgenic lettuce directly against hepatitis B virus (HBV), and initial results have shown that HBV-positive patients drunk the juice of this lettuce displayed significantly decreased levels of virus DNA titer and HbsAg (paper in revision).

One more issue I need to clarify clearly: The dietary microRNA we are discussing represents all types of exogenous small non-coding RNA. MIR2911 is certainly not a classic microRNA. However, its function is same to that of the endogenous animal microRNA in host cell (Zhou [37]). There are many reports that exogenous small non-coding RNA (not classic microRNA) functions as endogenous microRNA and plays important role in host cells We have also found that a Salmonella-encoded small RNA (70 nt) was processed to 21 nt RNA fragment and this small RNA fragment inhibited NOSi gene translation in mouse GI epithelial cells in a manner of an endogenous microRNA (paper in press).

Taken together, the extracellular RNA communication is a novel and important field which needs more investigators to study.

## Dependence on various forms of self-reports of dietary intake remains a serious challenge (due to potential for measurement error) for many in the field of nutrition, especially those seeking to evaluate potential links between specific foods and specified health outcomes. What is the potential usefulness of microRNAs as biomarkers of dietary intake (this could reflect endogenous as well as exogenous microRNA) or of functional responses to diet?

### Ken Witwer responds

Hypothetical miRNA markers of dietary intake could be endogenous or exogenous and would presumably be harvested from blood, urine, or feces. There is some evidence that endogenous miRNAs in different bodily compartments are modulated by dietary factors, such as glucose, vitamins, trace elements, drugs, or simply intake of food in general [52–54]. This modulation might occur through miRNA regulation in cells and/or by differential release from cells exposed to dietary components. Post-prandial shifts of circulating lipid particle populations, for example, would be reflected by miRNA if certain miRNAs were associated with specific lipoproteins. Specificity and timing issues challenge the development of endogenous miRNA markers of specific foods. First, miRNAs appear to be responsive to food components and breakdown products, not specific foods. A glucose-sensitive miRNA, for example, could not tell us if the donor ate an apple or used creamer in her coffee. Second, it is unlikely that any endogenous miRNA responds to dietary factors alone; abundant miRNAs have been proposed as markers of a wide variety of conditions and diseases but may be specific to none of them [55]. Third, many abundant animal miRNAs share 100% identity across species, such that endogenous upregulation cannot be distinguished from influx of xenomiRs [56]. Fourth, the timing and design of sampling is important. With a pre-prandial sample establishing a baseline, a post-prandial sample should be taken within a carefully established interval since RNAs are rapidly cleared from circulation, with a half-life of minutes to tens of minutes depending on protein and lipid associations. An endogenous miRNA-based assay would be informative just around the sampling window: unfortunately, this when alternative metrics are least needed due to accurate reporting of recent events or in-clinic monitoring pre- and post-prandially. Taken together, endogenous miRNAs may be found to act as reliable surrogate markers for intake of classes of dietary substances. However, high-performance blood assays are already available for these substances.

Exogenous miRNAs—if they differ in sequence from endogenous miRNAs—might offer a better opportunity for detection of specific foods, albeit again within a short period of time following intake. Although there is little convincing evidence that nucleic acids are absorbed from the diet in functionally relevant forms or amounts, and no well-established mechanisms for such transfer, even trace uptake could, theoretically, reveal dietary sources. But are miRNAs the best candidates? The ideal nucleic acid marker(s) would be stable, abundant, and information-rich, including specificity to the food of interest. From a stability perspective, the relative resistance of DNA to hydrolysis would recommend it over RNA, and ribonucleoprotein (RNP) complexes over free RNA. However, mechanisms for absorption of intact DNA and RNPs from the diet are not known. The concept of abundance is related to stability: at a given degree of stability, the more abundant molecule is more likely to survive the harsh trip through the alimentary canal and thus be available for hypothetical uptake. High copy number RNAs, such as tRNAs or rRNAs, are abundant but also highly conserved, requiring relatively large amounts of sequence to find discriminatory differences. Messenger RNAs number in the tens of thousands, are hundreds to thousands of bases long, and often undergo alternative splicing. In comparison, circulating xenomiRs are disadvantaged as markers. They are short (little information), highly conserved across species and within kingdoms, and not particularly diverse (only hundreds to thousands of predicted miRNAs per species, with only a handful highly expressed in any given cell type). A plant miRNA might reveal that a plant was ingested, or even if it was a dicot or a monocot. However, for many food items, complete atlases of miRNA sequences and expression levels are not yet reliable, nor do we know how processing affects the availability of miRNAs in tissue; other than that it is quite variable. Proposed species-specific miRNAs may be predicted in silico only, have atypical precursor structures, and be disputed as genuine miRNAs; in any case, the rule of thumb that conservation correlates with abundance suggests that low copy number, species-specific miRNAs would be difficult to detect. Assays sufficiently sensitive to detect rare plant miRNAs would be expensive and prone to the pervasive and confounding influence of contamination. Finally, different assays (or full sequencing) would be necessary to identify different foods, making screening difficult and expensive compared with verification of a single food item.

In summary, body fluid miRNAs seem to be poorly suited as markers of specific dietary intake. Circulating endogenous miRNAs may serve as general nutrition indicators but cannot discriminate between specific foods and are unlikely to reveal dietary history for more than a few hours. Endogenous miRNAs represent a drastically more expensive and complicated alternative to existing metabolite blood tests. In contrast, exogenous miRNAs may enter the blood at very low, non-functional levels and could be markers of recent intake of classes of foods such as plants or even subcategories within the plant kingdom. However, other types of RNA (or DNA) are likely to be more informative than short, highly conserved miRNAs. Research programs focused on the uptake of xenomiRs as biomarkers of dietary intake are based on false assumptions and faulty studies and would be better channeled towards improving subject monitoring or direct measurements of food-specific factors in feces.

### Chen-Yu Zhang responds

Although there is still no consensus on whether dietary miRNA can be categorized as a biomarker of dietary intake to date, the potential of dietary miRNA as a nutritional biomarker or biomarker of functional responses to diet is beyond doubt. Philip et al. [57] confirmed that dietary plant miRNAs are stably present in intact form after storage, processing, cooking, and early digestion in vivo. This study potentially indicates that dietary plant miRNAs have a robustness that makes them bioavailable for being used as a nutritional biomarker. Importantly, the correlations between specific dietary plant miRNAs and specific health outcomes have been established. We showed that plant miRNA is correlated with blood LDL levels [6, 7]. Chin et al. [19] confirmed that plant MIR159 is inversely correlated with breast cancer incidence and progression in patients. Cavalieri et al. [39] reported that plant miRNAs can serve as a novel form of immunomodulatory agents. These studies may shed light in future research of dietary miRNAs as new markers or components of nutrition.

For plant miRNAs serving as a biomarker of dietary intake, although it has been shown that plasma from an individual who reported following a vegetarian diet had a relatively high proportion of plant sequences [34], it is quite difficult to determine the exact plant foods consumed due to the conservation of miRNA sequences among different plant species. Thus, miRNA can serve as a biomarker of certain dietary state or health outcome rather than a direct reflection of specific food intake. Future studies in this area may screen dietary miRNAs for biomarkers of healthy and unhealthy eating habits.

### Ken Witwer concludes

Far from beyond doubt, dietary miRNAs as valid markers of (1) intake or (2) functional effects may be contradicted by the existing evidence and even the (albeit stimulating) arguments provided above. For the first—intake—a useful marker would reflect both the identity and dose of source material. Yet my interlocutors agree that the sequence conservation of miRNAs is incompatible with discrimination of specific food sources. Furthermore, they observe that apparent dietary miRNA absorption is not dependent on miRNA abundance in the source material. Indeed, in their study [6, 7], only four plant miRNAs (all highly conserved and abundant in plants) were detected in each of ten samples (each sample pooled from ten humans), with read counts varying from three to 28,000 [6, 7, 56]. Even if these reads were not consistent with contamination, as they are in [8], it is clear that with such tremendous variation, despite the moderating effects of pooling, they could not be used to identify the type or amount of food in the diet. For the second point—function—the Zhang group presents two studies of putative function [6, 7, 19]. The LDL study [6, 7] had a curious result, since eating raw plants is not usually associated with higher LDL levels; indeed, a more completely controlled reproduction study found that the increase was due to cholesterol mobilization in a starvation state—raw rice is nutritionally insufficient for rodents—not miRNA uptake or miRNA-mediated gene regulation, which was not detected [9, 58]. The Chin et al. study focused on minute levels of a xenomiR in blood that were well below standard limits of detection for miRNAs, including those used by the same group in a previous study [59] (averaging less than three copies per milliliter of blood). The very deep sequencing that would be required to detect such low levels of markers with confidence would be cost-prohibitive, not to mention that the subsequent animal study introduced exposure levels approximately 10^15^ times higher than what was observed in patient blood. To conclude, there is insufficient evidence for circulating xenomiRs as markers of dietary intake or response to diet. As previously stated, fecal miRNAs could be extracted from undigested (and thus unabsorbed) material, but other RNAs or DNA would provide better discrimination of dietary components.

## Footnotes

1 Reference 49 has been retracted by the authors. https://www.nature.com/articles/srep46826. The authors reported that "we were unable to confirm specific amplification of these miRNAs in human blood. Thus, we were not able to validate the central hypothesis of this paper."
